# Intraoperative Resection Guidance and Rapid Pathological Diagnosis of Osteosarcoma using B7H3 Targeted Probe under NIR‐II Fluorescence Imaging

**DOI:** 10.1002/advs.202310167

**Published:** 2024-03-19

**Authors:** Fanwei Zeng, Changjian Li, Han Wang, Yueqi Wang, Tingting Ren, Fangzhou He, Jie Jiang, Jiuhui Xu, Boyang Wang, Yifan Wu, Yiyang Yu, Zhenhua Hu, Jie Tian, Shidong Wang, Xiaodong Tang

**Affiliations:** ^1^ Department of Musculoskeletal Tumor & Beijing Key Laboratory of Musculoskeletal Tumor Peking University People's Hospital Beijing 100044 China; ^2^ School of Engineering Medicine & Key Laboratory of Big Data‐Based Precision Medicine Beihang University Ministry of Industry and Information Technology Beijing 100191 China; ^3^ CAS Key Laboratory of Molecular Imaging Beijing Key Laboratory of Molecular Imaging Institute of Automation Chinese Academy of Sciences Beijing 100190 China

**Keywords:** B7H3, intraoperative fluorescence‐guided surgery, near‐infrared imaging, osteosarcoma, targeted probe

## Abstract

Complete removal of all tumor tissue with a wide surgical margin is essential for the treatment of osteosarcoma (OS). However, it's difficult, sometimes impossible, to achieve due to the invisible small satellite lesions and blurry tumor boundaries. Besides, intraoperative frozen‐section analysis of resection margins of OS is often restricted by the hard tissues around OS, which makes it impossible to know whether a negative margin is achieved. Any unresected small tumor residuals will lead to local recurrence and worse prognosis. Herein, based on the high expression of B7H3 in OS, a targeted probe B7H3‐IRDye800CW is synthesized by conjugating anti‐B7H3 antibody and IRDye800CW. B7H3‐IRDye800CW can accurately label OS areas after intravenous administration, thereby helping surgeons identify and resect residual OS lesions (<2 mm) and lung metastatic lesions. The tumor‐background ratio reaches 4.42 ± 1.77 at day 3. After incubating fresh human OS specimen with B7H3‐IRDye800CW, it can specifically label the OS area and even the microinvasion area (confirmed by hematoxylin‐eosin [HE] staining). The probe labeled area is consistent with the tumor area shown by magnetic resonance imaging and complete HE staining of the specimen. In summary, B7H3‐IRDye800CW has translational potential in intraoperative resection guidance and rapid pathological diagnosis of OS.

## Graphical abstract

Herein, osteosarcoma (OS) targeting probe B7H3‐IRDye800CW is synthesized by conjugating anti‐B7H3 antibody and IRDye800CW. B7H3‐IRDye800CW could help surgeons identify and resect OS lesions after intravenous injection. After incubating the OS specimen with B7H3‐IRDye800CW, it could specifically label the OS invasion area, which is consistent with the tumor area shown by magnetic resonance imaging and hematoxylin‐eosin staining.

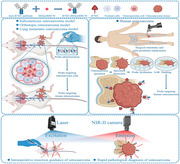

## 1 Introduction

Osteosarcoma (OS) is the most common primary bone tumor among children and adolescents.^[^ ^1^ ^]^ The unknown etiology, significant histological heterogeneity, lack of biomarkers, high aggressiveness, and early metastasis potential make OS a devastating disease, the 5‐year survival rate is 60% for patients with localized OS, but 20% for patients with metastases and OS recurrence,^[^ ^2^ ^]^ the improvement of OS survival rate has been plateaued for 3 decades,^[^ ^3^ ^]^ which indicates an urgent need for more effective treatment and diagnosis methods.^[^ ^4^ ^]^ Complete surgical resection of OS remains essential for patient survival.^[^ ^5^ ^]^ The incidence of local recurrence was reported to be closely related to the achieved surgical margins.^[^ ^6^ ^]^ Currently, surgeons can only rely on limited visual and tactile information combined with personal experience to distinguish tumor lesions and normal tissues,^[^ ^7^ ^]^ which is subjective and inaccurate.^[^ ^8^ ^]^ Overestimation of tumor boundary leads to unnecessary tissue resection and enormous loss of function postoperatively, while underestimation can lead to tumor residuals, leading to local recurrence and worse prognosis.^[^ ^9^ ^]^ Besides, the value of frozen‐section analysis is limited for OS surgery because it is time‐consuming and sometimes inaccurate due to sampling errors, the hard tissues in OS‐prone areas (distal femur, proximal tibia, and proximal humerus) also greatly restrict the frozen‐section analysis,^[^ ^10^ ^]^ routine decalcification and subsequent pathological analysis could take several days and cannot meet the needs of intraoperative diagnosis, the present technical bottleneck makes it impossible for surgeons to know intraoperatively whether a negative margin has been achieved. Therefore, a method that can instantly and reliably help surgeons distinguish tumors from healthy tissues and assess surgical margins is urgently needed.

Near‐infrared (NIR) fluorescence imaging has shown unique advantages in intraoperative tumor resection guidance,^[^ ^11^ ^]^ it provides tumor‐related fluorescence information intraoperatively in a non‐radiative and non‐invasive manner. Nowadays, several fluorescent probes have been approved by Food and Drug Administration of the United States for fluorescence‐guided tumor resection, such as 5‐ALA^[^ ^12^ ^]^ and OTL38,^[^ ^13^ ^]^ a large number of fluorescent probes have been tested in clinical trials^[^ ^14^ ^]^ including indocyanine green (ICG),^[^ ^15^ ^]^ Bevacizumab‐IRDye800CW,^[^ ^16^ ^]^ Panitumumab‐IRDye800CW,^[^ ^17^ ^]^ SGM‐101,^[^ ^18^ ^]^ and EC17.^[^ ^19^ ^]^ NIR probe ICG has been used in the resection guidance of OS in clinical trials, however, our previous study has shown that ICG, the most commonly used fluorescent probe, has only an accuracy of 19% in detecting tumor residuals after en bloc resection of skeletomuscular malignancies and is not suitable for the guidance of wide resection of OS,^[^ ^15a^ ^]^ it is because ICG accumulates in tumors by enhanced permeability and retention effect rather than tumor cell‐specific binding.^[^ ^20^ ^]^ Besides, no targeting fluorescent probe has been used in the clinical trials of OS, which highlights the need for an OS‐targeting probe.

Recently, the researchers gradually shifted their attention from the conventional NIR window (750–900 nm, NIR‐I) to the second NIR window (1000–1700 nm, NIR‐II). Less light scattering, tissue autofluorescence, and photon absorption at the NIR‐II window greatly improve tumor‐to‐background ratio, spatial resolution, and penetration depth.^[^ ^21^ ^]^ The application of NIR‐II fluorescence will greatly improve the accuracy of tumor resection.^[^ ^22^ ^]^ To the best of our knowledge, using NIR‐II fluorescence imaging for intraoperative resection guidance and rapid pathological diagnosis of OS has not been reported yet.

B7H3 is an immune checkpoint transmembrane protein with a length of 316 amino‐acid, B7H3 is overexpressed in differentiated malignant cells and cancer‐initiating cells and many tumor types (60% of 25 000 tumor samples),^[^ ^23^ ^]^ while B7H3 has a low expression in normal tissues.^[^ ^24^ ^]^ Overexpression of B7H3 is closely related to tumor progression, metastasis, and poor clinical outcome.^[^ ^25^ ^]^ Currently, many B7H3‐based clinical trials are ongoing including B7H3‐based chimeric antigen receptor T‐cell immunotherapy and anti‐B7H3 antibody‐dependent cellular cytotoxicity.^[^ ^26^ ^]^ Furthermore, there are already evidences supporting the high expression of B7H3 in OS,^[^ ^24^, ^27^ ^]^ making B7H3 an excellent target candidate for OS.

In this study, in order to solve the current difficulties in OS surgery, we took advantage of the high expression of B7H3 in OS. An anti‐B7H3 antibody was conjugated with NIR fluorescent dye IRDye800CW to construct an OS‐targeting NIR‐II probe B7H3‐IRDye800CW, the intraoperative resection guidance and rapid pathological diagnosis value of B7H3‐IRDye800CW under NIR‐II fluorescence imaging was systematically evaluated.

## 2 Results

### 2.1 B7H3 Expression among OS Tissues/Cells and Normal Tissues/Cells

**Figure 1:**
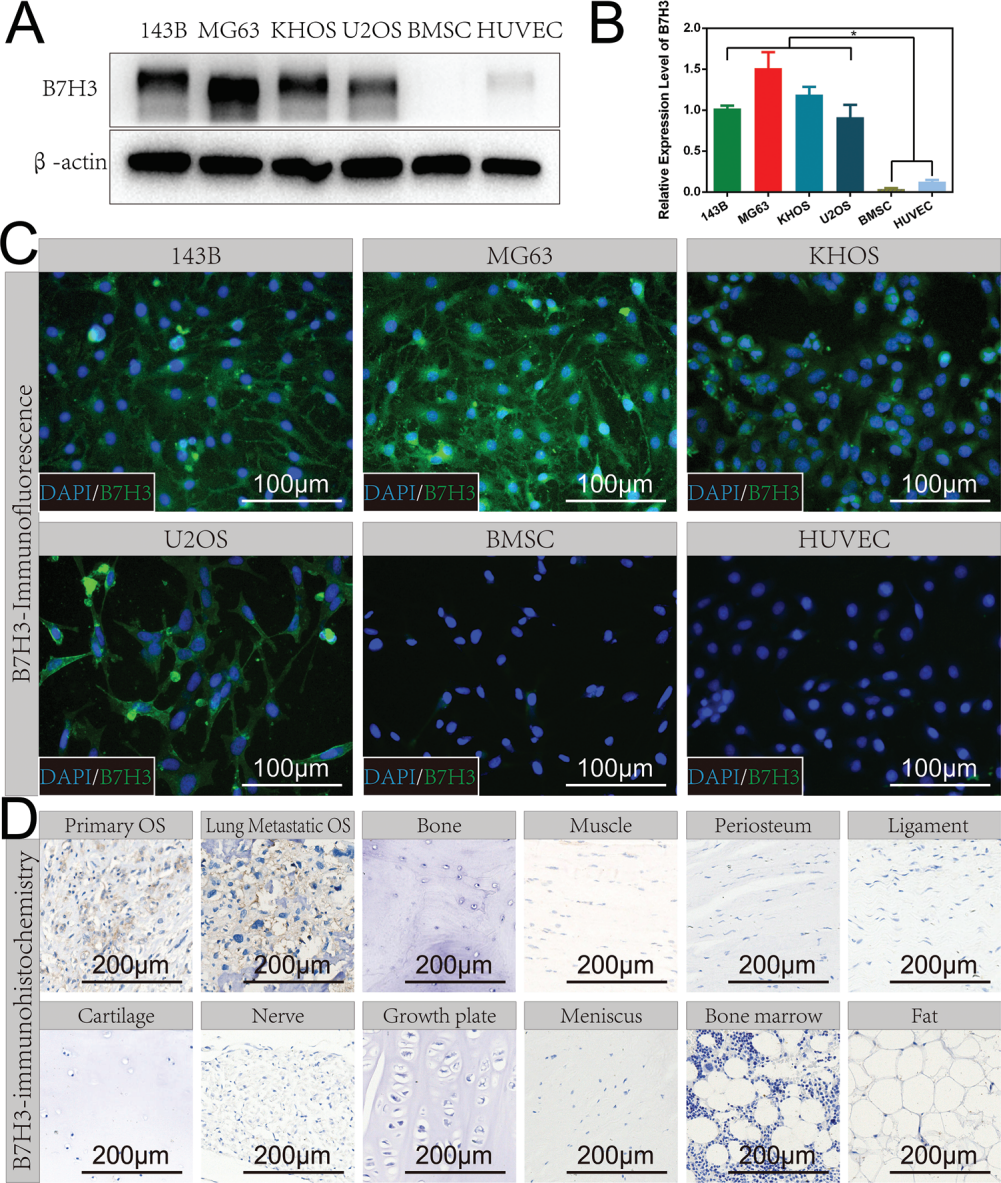
Verification of B7H3 target in OS. A) Western‐blot results of B7H3 expression in osteosarcoma cell lines (143B, MG63, KHOS, U2OS) and normal cells (BMSC, HUVEC). B) Quantitative data of B7H3 expression. C) B7H3 immunofluorescence images of osteosarcoma cell lines (143B, MG63, KHOS, U2OS) and normal cells (BMSC, HUVEC). D) B7H3 immunohistochemical images of human primary OS, lung metastatic OS, and common normal tissues in OS‐prone areas (bone, muscle, periosteum, ligament, cartilage, nerve, growth plate, meniscus, bone marrow, and fat). Scale bars are shown in the figure.

To explore and verify the feasibility of B7H3 as a promising OS target, we first evaluated the expression of B7H3 among four OS cell lines (143B, MG63, KHOS, and U2OS) and two normal cells (bone marrow‐derived mesenchymal stem cells, BMSC and human umbilical vein endothelial cells, HUVEC). The western‐blot results showed that four OS cell lines had significantly higher B7H3 expression than normal cells (**Figure** **1**A,B). The immunofluorescence results further confirmed that B7H3 was highly expressed in OS cell lines, while the expression of B7H3 in normal cells is very low (Figure 1C and Figure S1, Supporting Information).

To further confirm the intraoperative guidance value of B7H3 for OS targeting, the expression of B7H3 was evaluated in human OS specimens (human primary OS and lung metastatic OS) and ten kinds of human normal tissues that commonly located around OS (bone, muscle, periosteum, ligament, cartilage, nerve, growth plate, meniscus, bone marrow, and fat). The results showed that the expression of B7H3 in human primary OS and lung metastatic OS was significantly higher than that of normal tissues in OS‐prone areas (Figure 1D and Figure S2, Supporting Information), thus, the significant difference in B7H3 expression between OS and surrounding normal tissues made it an excellent OS target.

### 2.2 Characterization and Cytotoxicity of B7H3 Targeted Fluorescent Probe

**Scheme 1:**
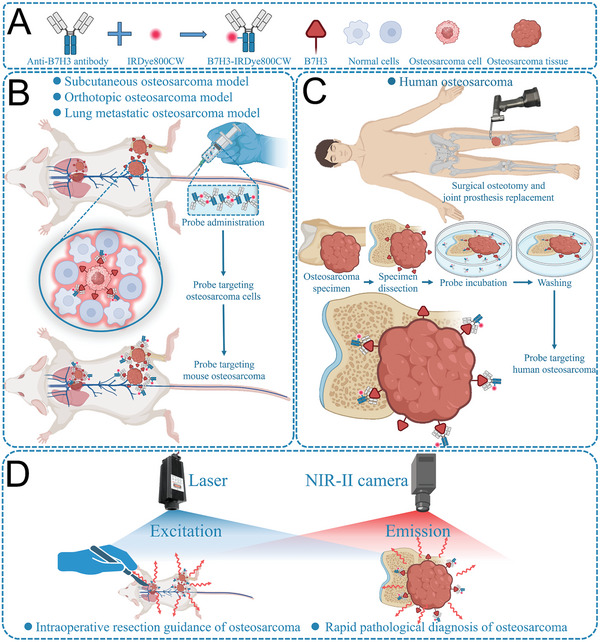
Schematic diagram of the whole study. A) Synthesis of B7H3‐IRDye800CW probe, schematic diagram of B7H3 target, normal cells, osteosarcoma cell, and osteosarcoma tissue. B) Establishing mouse subcutaneous osteosarcoma model, orthotopic osteosarcoma model, and lung metastatic osteosarcoma model, after intravenous injection of B7H3‐IRDye800CW, B7H3‐IRDye800CW could bind osteosarcoma cells with high expression of B7H3, thus targeting mouse osteosarcoma. C) For osteosarcoma patients receiving osteotomy of tumor bones, the specimens were sawed into 3 mm thickness, incubated in B7H3‐IRDye800CW, and washed to remove the nonspecific binding probe. D) NIR‐II guided intraoperative resection guidance and rapid pathological diagnosis of osteosarcoma.

**Figure 2:**
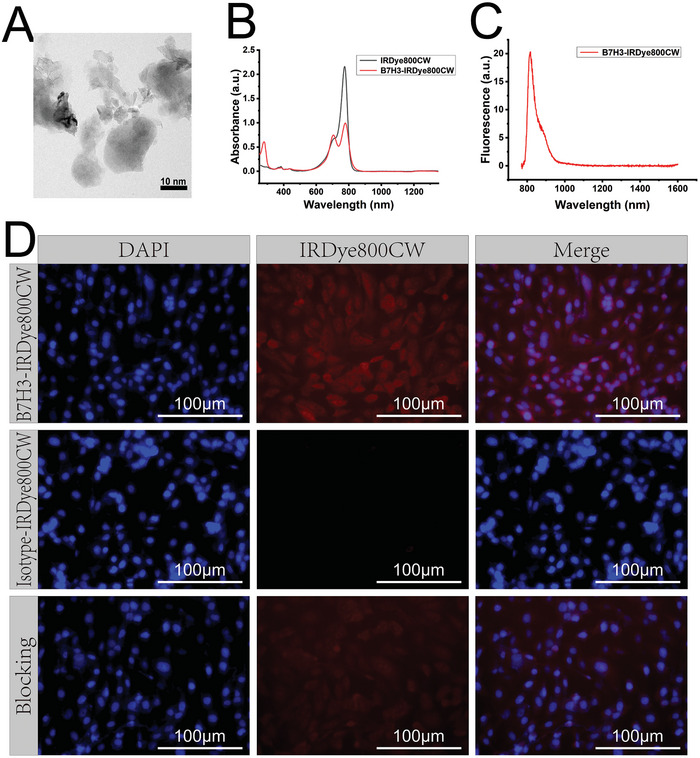
B7H3‐IRDye800CW characterization and in vitro targeting ability evaluation. A) Transmission electron microscopy of B7H3‐IRDye800CW. B) The UV–vis‐NIR absorption spectrum of IRDye800CW and B7H3‐IRDye800CW. C) The fluorescence spectrum of B7H3‐IRDye800CW. D) Immunofluorescence staining of 143B by B7H3‐IRDye800CW, isotype‐IRDye800CW, and block experiment. Scale bar: 100 µm.

B7H3‐IRDye800CW was synthesized by conjugating IRDye800CW NHS Ester and Anti‐B7H3 antibody (**Scheme** **1**A). The transmission electron microscopy image of B7H3‐IRDye800CW showed that B7H3‐IRDye800CW has an average diameter of 20 nm (**Figure** **2**A). B7H3‐IRDye800CW had an absorption peak at 280 nm while IRDye800CW did not (Figure 2B), the characteristic absorption peak of proteins meant that IRDye800CW was successfully conjugated to anti‐B7H3 antibody. B7H3‐IRDye800CW had a maximum emission wavelength at 810 nm and a long NIR‐II emission tail that extended to 1500 nm (Figure 2C). The average number of IRDye800CW conjugated to one anti‐B7H3 antibody was 1.77 (Figure S3, Supporting Information). Both the B7H3‐IRDye800CW probe and the anti‐B7H3 antibody have high affinity for the B7H3 target, the conjugation of IRDye800CW to anti‐B7H3 antibody only slightly reduced the affinity of the anti‐B7H3 antibody. B7H3‐IRDye800CW had good biocompatibility even at 256 µg mL^−1^, and the cytotoxicity of the B7H3‐IRDye800CW probe was lower than ICG at the same concentration (*p* < 0.05, Figure S4, Supporting Information).

### 2.3 In Vitro Targeting Ability Evaluation

143B cells were incubated with B7H3‐IRDye800CW and isotype‐IRDye800CW, the results showed that B7H3‐IRDye800CW could bind to 143B (Figure 2D), while isotype‐IRDye800CW cannot. Besides, after blocking by a high dose of anti‐B7H3 antibody and followed by incubation with B7H3‐IRDye800CW, the fluorescence signal was weaker compared with the group without the blocking step, this was because a large number of B7H3 targets were pre‐occupied by anti‐B7H3 antibody.

### 2.4 In Vitro and In Vivo Comparison of NIR‐I and NIR‐II Fluorescence Imaging

**Figure 3:**
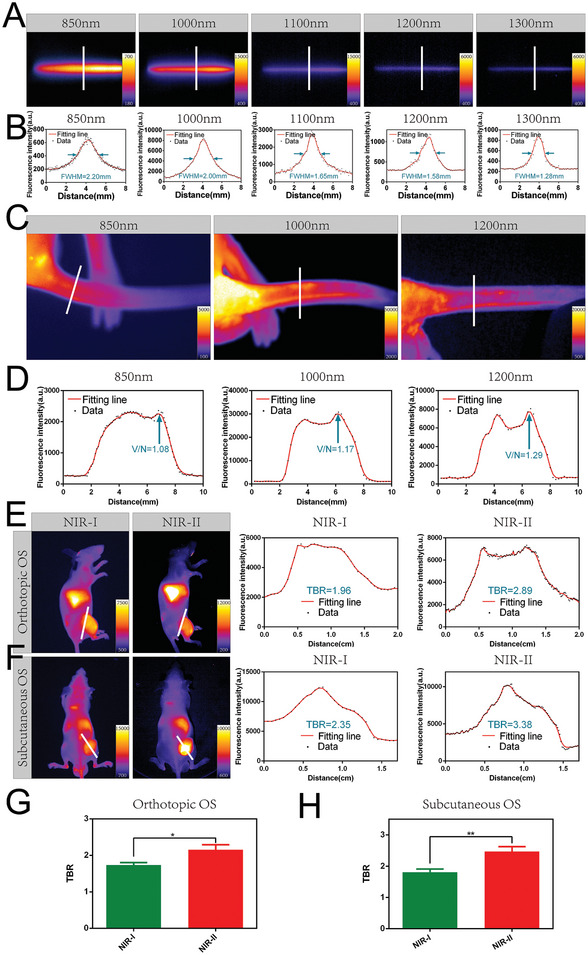
Comparison of NIR‐I/II fluorescence imaging in vitro and in vivo. A) NIR fluorescence imaging of the capillary tube filled with B7H3‐IRDye800CW at 850, 1000, 1100, 1200, and 1300 nm. B) LOWESS smoothing of fluorescence intensity of the white lines in (A), the FWHM was 2.20, 2.00, 1.65, 1.58, and 1.28 mm at 850, 1000, 1100, 1200, and 1300 nm, respectively. C) Mouse tail vessel fluorescence imaging at 850, 1000, and 1200 nm. D) LOWESS smoothing of fluorescence intensity of the white lines in (C), the V/N was 1.08, 1.17, and 1.29 at 850, 1000, and 1200 nm, respectively. E) NIR‐I and NIR‐II fluorescence imaging of orthotopic OS, LOWESS smoothing of fluorescence intensity of the white lines. F) NIR‐I and NIR‐II fluorescence imaging of subcutaneous OS, LOWESS smoothing of fluorescence intensity of the white lines. TBR of NIR‐I and NIR‐II fluorescence images of G) orthotopic OS and H) subcutaneous OS. (* *p* < 0.05, ** *p* < 0.01).

To verify the NIR‐II imaging advantage of B7H3‐IRDye800CW, a capillary tube filled with B7H3‐IRDye800CW was placed in fat emulsion, and NIR imaging was then conducted at different wavelengths (850–1300 nm). The most severe scattering was observed at the NIR‐I window (850 nm), while at the NIR‐II window (1000–1300 nm), the light scattering was significantly less (**Figure** **3**A). The full width at half maxima (FWHM) was 2.20, 2.00, 1.65, 1.58, and 1.28 mm at 850, 1000, 1100, 1200, and 1300 nm (Figure 3B), respectively. For mouse tail vessel imaging, NIR‐I imaging (850 nm) could only reveal the vessel vaguely (Figure 3C), owing to more light scattering and less penetration depth of NIR‐I imaging. The tail vessel was clearer in the NIR‐II window (1000 and 1200 nm), which means higher spatial resolution. The ratio of the fluorescence signal of blood vessels to the fluorescence signal of surrounding normal tissue (V/N) was 1.08, 1.17, and 1.29 at 850, 1000, and 1200 nm, respectively (Figure 3D). Long wavelength can reduce scattering and improve the spatial resolution of deep tissue, but meanwhile, the decrease in absolute fluorescence intensity should also be noticed, therefore, a balanced wavelength (1000 nm) was selected in the subsequent NIR‐II fluorescence imaging. In the mouse subcutaneous and orthotopic OS model, the same mice were imaged under NIR‐I and NIR‐II, it could be found that the background fluorescence signal is darker and the tumor is more distinguishable under NIR‐II imaging (Figure 3E,F). NIR‐II imaging of B7H3‐IRDye800CW can bring higher tumor‐background ratio (TBR) (orthotopic model: 2.16 ± 0.31; subcutaneous model: 2.47 ± 0.34) in mouse subcutaneous and orthotopic OS model compared with NIR‐I (orthotopic model: 1.73 ± 0.15; subcutaneous model: 1.81 ± 0.23) (Figure 3G,H).

### 2.5 The Relationship between the Concentration of B7H3‐IRDye800CW and NIR‐I/II Fluorescence Intensity

The fluorescence intensity of B7H3‐IRDye800CW at different concentrations (2, 4, 6, 8, 10 and 12 µg mL^−1^) under NIR‐I and NIR‐II imaging was recorded (Figures S5 and S6, Supporting Information). The NIR‐I and NIR‐II fluorescence intensity of B7H3‐IRDye800CW is linearly related to the concentration (R^2^ = 0.99 at NIR‐I, R^2^ = 0.95 at NIR‐II).

### 2.6 In Vivo Targeting Ability and Biodistribution Evaluation

**Figure 4:**
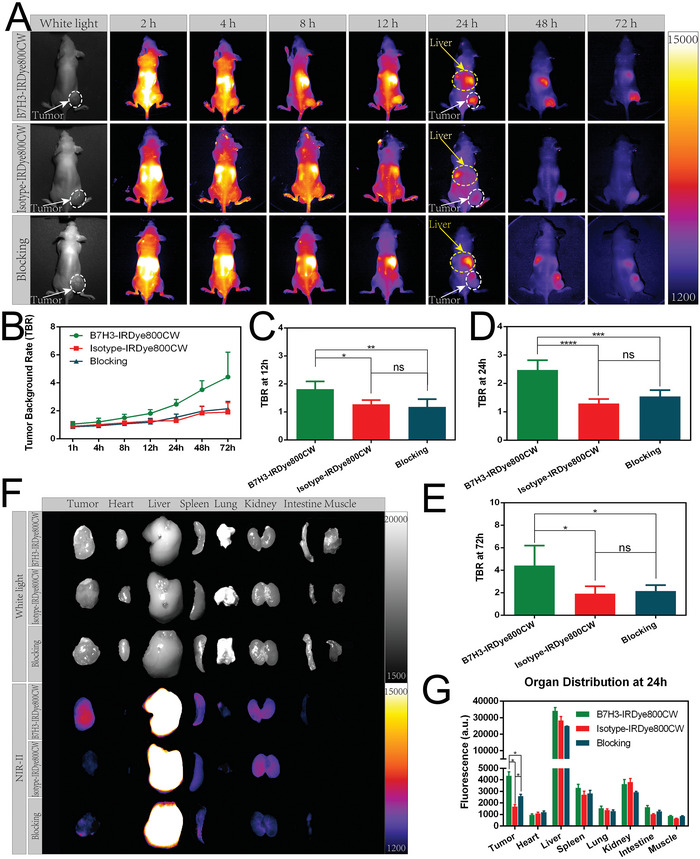
In vivo targeting ability and organ distribution of B7H3‐IRDye800CW. A) Representative fluorescent images of B7H3‐IRDye800CW, isotype‐IRDye800CW, and blocking group at 2, 4, 8, 12, 24, 48, and 72 h after administration of probe, the areas indicated by yellow arrows were livers, areas indicated by white arrows were tumors. B) TBR of B7H3‐IRDye800CW, isotype‐IRDye800CW, and blocking group under NIR‐II fluorescence imaging. TBR at C) 12 h, D) 24 h, and E) 72 h. F) In vivo organ distribution of B7H3‐IRDye800CW, isotype‐IRDye800CW, and blocking group at 24 h. G) NIR‐II fluorescence intensity of tumor and major organs in three groups.

The in vivo targeting ability was evaluated in the mouse subcutaneous OS model (**Figure** **4**A–E). The liver showed a strong fluorescence signal and the fluorescence signal gradually decreased with time. In the B7H3‐IRDye800CW group, the fluorescence signal could be observed in the tumor site 2 h after probe administration. At 8 h, the tumors were well differentiated from surrounding normal tissue and became more recognizable with time. TBR continued to increase during the whole observation period (TBR = 1.81 ± 0.28 at 12 h, TBR = 2.47 ± 0.34 at 24 h, TBR = 4.42 ± 1.77 at 72 h). In the isotype‐IRDye800CW group, the tumors could be hardly distinguished from surrounding normal tissue during the whole observation period (TBR = 1.27 ± 0.15 at 12 h, TBR = 1.30 ± 0.15 at 24 h, TBR = 1.91 ± 0.67 at 72 h). In the blocking group, tumors are also indistinguishable from normal tissue (TBR = 1.18 ± 0.27 at 12 h, TBR = 1.54 ± 0.23 at 24 h, TBR = 2.16 ± 0.52 at 72 h). At 12, 24, and 72 h, the B7H3‐IRDye800CW group showed higher TBR than the isotype‐IRDye800CW group and the blocking group (*p* < 0.05), and B7H3‐IRDye800CW was almost fully metabolized on the seventh day (Figure S7, Supporting Information).

In in vivo biodistribution experiment, it was found that for B7H3‐IRDye800CW, isotype‐IRDye800CW, and blocking group, the fluorescence was strongest in the liver, which indicated that B7H3‐IRDye800CW and isotype‐IRDye800CW are mainly metabolized by liver (Figure 4F,G), the remarkable difference among three groups is that B7H3‐IRDye800CW group had more probe distribution in the tumor than the other two groups (*p* < 0.05), the HE staining of major organs in three groups did not find obvious toxicity, which meant that intravenous injection of B7H3‐IRDye800CW will not cause organ toxicity (Figure S8, Supporting Information).

### 2.7 In Vivo Targeting Accuracy Evaluation

**Figure 5:**
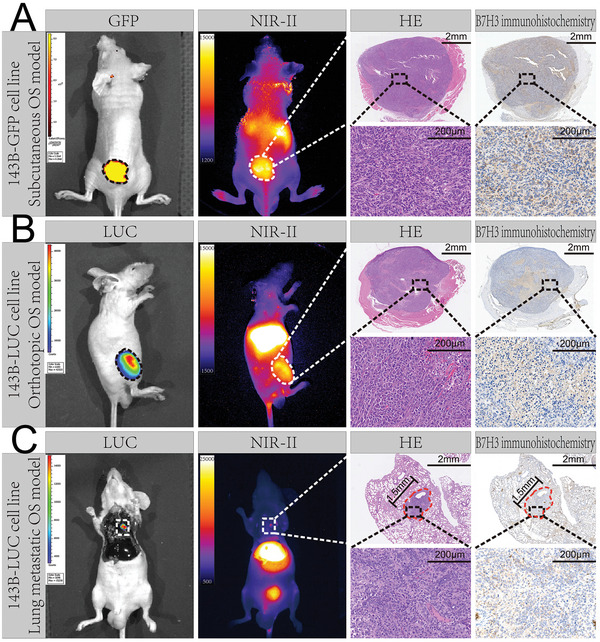
Targeting accuracy evaluation of B7H3‐IRDye800CW. Targeting accuracy evaluation of B7H3‐IRDye800CW in A) mouse subcutaneous OS model, B) mouse orthotopic OS model, and C) mouse lung metastatic OS model. GFP fluorescence imaging and bioluminescence (BLI) imaging were used to show the actual tumor areas. Low magnification and high magnification images of HE staining and B7H3 immunohistochemical staining of the tumors are shown on the right. Scale bars are labeled on the figure.

The OS targeting accuracy of the B7H3‐IRDye800CW probe was evaluated by whether the probe can selectively label the tumor area. It could be observed that the areas labeled by B7H3‐IRDye800CW and the areas labeled by green fluorescent protein (GFP) and luciferase (LUC) (**Figure** **5**A–C) have exactly the same shape and position. Subsequently, the tissues lighted by B7H3‐IRDye800CW were dissected for pathological analysis, HE staining and B7H3 immunohistochemistry all proved that the resected tissues were OS.

### 2.8 NIR‐II Fluorescence Imaging Guided OS Resection

**Figure 6:**
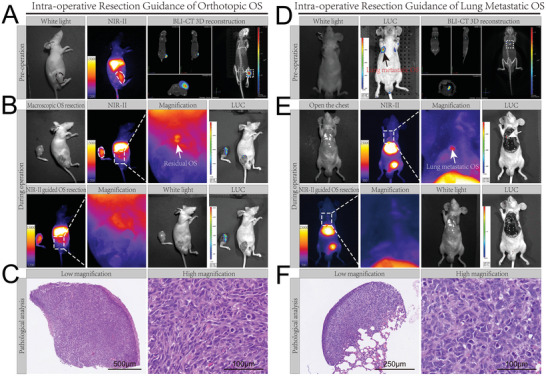
Intraoperative resection guidance of orthotopic OS and lung metastatic OS. A) Orthotopic OS localization by NIR‐II fluorescence and BLI‐CT 3D reconstruction imaging. B) NIR‐II fluorescence‐guided resection of mouse orthotopic OS. C) HE staining of excised tissue in mouse orthotopic OS model. D) Location of lung metastatic OS by BLI and BLI‐CT 3D reconstruction imaging. E) NIR‐II fluorescence‐guided resection of mouse lung metastatic OS. F) HE staining of excised tissue in mouse lung metastatic OS model. Scale bars are labeled on the figure.

Complete resection of the tumor is very important in the treatment of OS, however, due to the small satellite lesions invisible to the naked eye and blurry tumor boundaries, as well as the narrow surgical field and blood stain in the surgical area, it is difficult to achieve. The feasibility of using the B7H3‐IRDye800CW probe in intraoperative resection guidance of OS was evaluated. For the mouse orthotopic OS model, NIR‐II, BLI‐CT 3D reconstruction imaging was conducted to localize the tumor (**Figure** **6**A), and the orthotopic OS was resected until no tumor could be found with the naked eye. Then, NIR‐II imaging was used to find residual OS, three of the five mice (60%) had high fluorescence signals in the surgical field (Figure 6B), subsequently, BLI imaging also found the same area with high signals, which confirmed that the areas were tumor residuals. Finally, the tumor residuals (<2 mm) were resected under the guidance of NIR‐II imaging (Figure 6B), BLI imaging was performed again and the result proved that all the tumor residuals were completely removed. For the mouse lung metastatic OS model, first, BLI and BLI‐CT 3D reconstruction imaging indicated a metastasis in the lung (Figure 6D). Then, the chest was opened to search for lung metastasis, which was hard to find with the naked eye (Figure 6E). Second, with the assistance of NIR‐II imaging, an area with a high fluorescence signal was located, the same area also emits BLI, which was proven to be OS, finally, the lung metastasis was resected under the guidance of NIR‐II imaging and BLI imaging proved that there was no tumor remains (Figure 6E). The excised areas with high fluorescence signals were all proved to contain OS by HE staining (Figure 6C,F).

### 2.9 NIR‐II Imaging of Human OS Specimens using B7H3‐IRDye800CW

**Figure 7:**
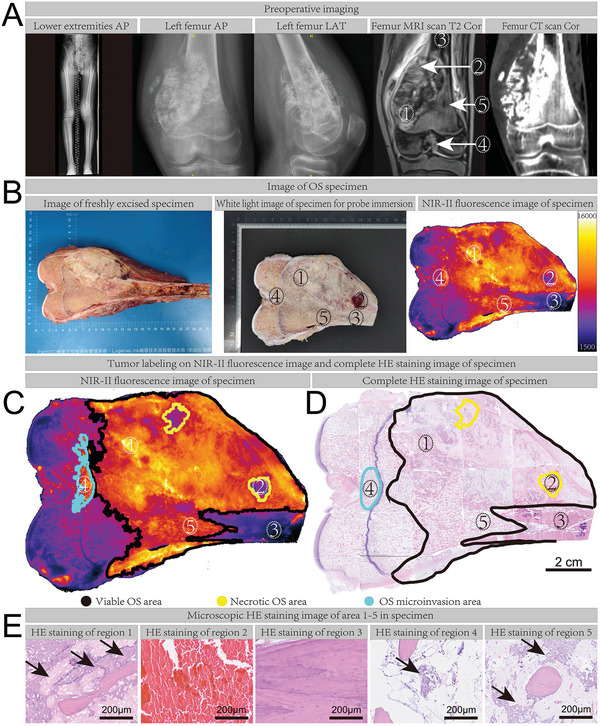
Rapid pathological diagnosis of human OS specimen. A) Preoperative digital radiography images of lower extremities anterior‐posterior, left femur anterior‐posterior, and left femur lateral position, T2‐weighted coronal MRI image of the left femur, coronal CT image of the left femur. B) Fresh resected specimen (already coronally resected), 3 mm thick specimen, and corresponding NIR‐II fluorescence image. C) NIR‐II fluorescence image with a label of OS areas. D) Complete HE staining of the specimen, the area circled by the black line is the viable OS area, the area circled by the yellow line is the necrotic OS area, and the area circled by the blue line is the OS microinvasion area. Scale bar: 2 cm. E) Microscopic HE staining pictures of five dashed number ellipses marked areas in MRI, white light, NIR‐II, and complete HE staining image, region 1 was viable OS, region 2 was necrotic OS area, region 3 was normal cortical bone, region 4 was OS microinvasion, region 5 was viable OS, the areas indicated by the black arrows were OS. Scale bar: 200 µm.

The general information of enrolled patients is shown in **Table** **1**. The segmentation method for the human OS specimen method is shown in Figure S9, Supporting Information. The experiment procedure is shown in Scheme 1C,D. The preoperative X‐ray, computed tomography (CT), and T2‐weighted MRI images of a representative OS patient (No. 4) were shown (**Figure** **7**A). After surgical excision, the specimen was further sawed into a 3 mm thickness slice (Figure 7B), and NIR‐II fluorescence imaging was performed after B7H3‐IRDye800CW soaking and washing steps. A strong fluorescence signal could be observed in the black line circled area (Figure 7C), the fluorescence intensity of adjacent tissue is weak, including growth plate, articular cartilage, ligament, normal cortical bone near the bone shaft, and normal cancellous bone near the joint, which was consistent with the previous B7H3 immunohistochemistry results. Next, HE staining was performed for the entire specimen to understand the tissue composition of the fluorescence‐highlighted area. The pathological tumor invasion area was outlined with black on a high‐magnification HE staining image (Figure 7D), we could see that the fluorescently highlighted tumor area was almost consistent with the tumor area verified by pathological evidence. Region 1 with a high fluorescence signal on NIR‐II imaging and abnormal mixed signal on T2‐weighted MRI imaging was proven to be viable OS by HE staining (Figure 7E). Region 5 has a high fluorescence signal on NIR‐II imaging but no significant abnormal change on T2‐weighted MRI imaging was also proved to contain viable tumor cells. Region 3 with low fluorescence signal on NIR‐II and normal signal on T2‐weighted MRI imaging turned out to be normal cortical bone.

**Table 1:** The general information of patients in the in vitro human OS specimen incubation and imaging experiment.

Patient No.	Age [year]	Gender	Location	Tumor size [cm]
1	15	Male	Left distal femur	12 × 9
2	12	Male	Right distal femur	5 × 5
3	13	Female	Left proximal humerus	9 × 5
4	13	Male	Left distal femur	11 × 8
5	11	Male	Left proximal femur	6 × 6

In this patient, preoperative MRI showed that the epiphysis was adjacent to the tumor and the growth plate was intact, MRI and CT cannot tell whether the epiphysis was invaded by the tumor. NIR‐II imaging of the resected sample showed high fluorescence signals (blue line circled and region 4 in Figure 7C) in this suspicious area. We further searched the area on HE staining and found multiple OS microinvasion lesions in this area (Figure 7E and Figure S10, Supporting Information), suggesting that the expression of B7H3 is elevated even in microinvasion areas of OS. In the black line circled area, we found some areas with low fluorescence signal (yellow line circled and region 2 in Figure 7C), region 2 turned out to be a necrotic OS area on HE staining (Figure 7E).

## 3 Discussion

As fluorescence‐guided surgery enters clinical practice rapidly, new targeted fluorescent probes designed for tumor resection guidance become research hot‐spots.^[^ ^28^ ^]^ Some targeted fluorescent probes have been tested in clinical trials, such as bevacizumab‐IRDye800CW, for breast and colorectal cancer surgery guidance.^[^ ^16^, ^29^ ^]^ OS is a devastating and rare disease with a poor prognosis, the limb‐sparing surgery of OS is challenging because there seems to be a contradiction between achieving a wide surgical margin and preserving normal tissue.^[^ ^30^ ^]^ However, to our knowledge, no targeted fluorescent probe designed for OS resection guidance has been reported, suggesting that the development of targeted fluorescent probes for OS resection guidance is urgent.

In this study, we developed an OS‐targeting NIR‐II probe B7H3‐IRDye800CW and evaluated its potential in intraoperative resection guidance and rapid pathological diagnosis. The in vivo and in vitro results showed that I) B7H3 was highly expressed in OS, while the expression was extremely low in various normal tissues in OS‐prone areas. The huge difference in B7H3 expression proved the feasibility of B7H3‐targeted strategy in OS; II) B7H3‐IRDye800CW had excellent NIR‐II imaging capabilities, NIR‐II imaging can bring higher spatial resolution, smaller tissue scattering, and higher in vivo imaging TBR; III) B7H3‐IRDye800CW had a wide time window for NIR‐II fluorescence‐guided surgery, 24–72 h after intravenous injection was the suitable time for resection guidance of OS; IV) B7H3‐IRDye800CW achieved accurate labeling of OS and was able to guide the resection of small residual OS lesions (<2 mm) and lung metastatic OS; V) B7H3‐IRDye800CW could realize intraoperative pathological diagnosis of OS by soaking the resected specimen, the area highlighted by B7H3‐IRDye800CW was almost consistent with the tumor area shown by pathology and MRI. It can also label the microinvasion area of OS, which was hard to find even on HE staining.

In previous studies, researchers found that B7H3 is a potential OS marker with limited expression in normal tissues.^[^ ^24b^ ^]^ However, they did not clarify the tissue types with low expression of B7H3, which was insufficient for B7H3 to be used as a target for resection guidance of OS. In this study, we investigated ten types of normal tissue that are commonly located in the surgical field of OS. The chosen ten types of normal tissue have a low expression of B7H3 compared to the high expression in OS. Therefore, the B7H3 targeting strategy is expected to provide good contrast in the surgical field.

NIR‐II fluorescence imaging has less light scattering, tissue autofluorescence, and photon absorption.^[^ ^31^ ^]^ A lot of NIR‐II fluorescent probes have been reported with excellent imaging performance,^[^ ^32^ ^]^ including donor‐acceptor‐donor fluorophores,^[^ ^33^ ^]^ xanthenes,^[^ ^34^ ^]^ and borondipyrromethanes.^[^ ^35^ ^]^ However, their translational ability is limited by unknown toxicology and immunogenicity.^[^ ^21d^ ^]^ The insufficient clinical application potential of NIR‐II fluorophores has prompted the search for alternatives. Although the maximum emission peak of IRDye800CW falls at 810 nm (NIR‐I), IRDye800CW has a long emission tail that extends to 1500 nm (NIR‐II), opening up a new way for clinical NIR‐II imaging. Our study confirmed that NIR‐II fluorescence imaging of B7H3‐IRDye800CW could bring higher TBR, spatial resolution, and penetration depth compared with NIR‐I fluorescence imaging, which is beneficial for deep tissue and blood vessel imaging. We also noted that a longer exposure time is needed to capture the fluorescence of B7H3‐IRDye800CW at a longer wavelength, for example, the exposure time is 30 ms at 1000 nm, while the exposure needs to be adjusted to 1 s at 1200 nm, the long exposure time will lead to low fluency of real‐time navigation video.

The cytotoxicity of B7H3‐IRDye800CW was lower than ICG at the same concentration, HE staining of mice major organs did not show obvious organ toxicity of B7H3‐IRDye800CW. Based on the results above, a reasonable assumption could be made that B7H3‐IRDye800CW is safe to use in humans.

In the mouse experiment, after the injection of B7H3‐IRDye800CW, the probe targeted the OS with blood circulation and the TBR increased gradually. Within 24 h after probe administration, the background signals were too strong to identify tumors from healthy tissue. As time passed, the clearance of non‐specific binding probes greatly reduced the background fluorescence signal, and the TBR kept rising. However, the absolute fluorescence intensity is decreasing over time, which means a longer exposure time and lower refresh rate. At 3–7 days, fluorescence intensity is too weak for intraoperative guidance, and the probe is nearly fully metabolized at 7 days. To balance the decline of fluorescence signal intensity and the ascent of TBR, we think the best time window for intraoperative resection guidance is 24–72 h after administration. The wide fluorescence imaging window (24–72 h) provides flexibility for probe injection and surgical scheduling, which also indicates good circulation stability. The strong fluorescence signal of the liver indicates that B7H3‐IRDye800CW is mainly metabolized by the liver. Strong fluorescence of the liver may interfere with the intraoperative guidance of OS in mice, however, that is not a problem because the surgical field of OS is far away from the liver in humans.

B7H3‐IRDye800CW showed excellent capacity in the resection guidance of OS. Our results of NIR‐II fluorescence‐guided resection indicated that B7H3‐IRDye800CW‐based NIR‐II imaging can identify and then guide the resection of small lesions (less than 2 mm) in the background of healthy tissue, the situation often occurs in the limb‐sparing surgery of OS patients. The capacity is promising for it may help surgeons decide which structure can be preserved and which should be removed, achieving precise resection of OS with lower local recurrence and a better postoperative function.^[^ ^36^ ^]^

B7H3‐IRDye800CW also showed potential in rapid pathological examination. Intraoperative frozen‐section is an important method to determine whether there is residual tumor tissue at the surgical margin. However, the intraoperative frozen section of OS is always limited by the hard tissue around OS, which makes it difficult to determine the OS surgical margin. In this study, the resected specimen of OS was immersed by B7H3‐IRDye800CW, and a NIR‐II fluorescence image was captured. The tumor area shown by NIR‐II fluorescence was compared with the area shown by MRI, which was the most important imaging tool for identifying the boundary of OS.^[^ ^6^ ^]^ It was found that the tumor area shown by the two methods was nearly identical. However, the adjacent planes of the MRI may be 10 mm apart, and the NIR‐II imaging plane may not match any of the planes in the MRI. As a solution, HE staining of the whole specimen was performed, we also found that the NIR‐II fluorescence labeled area was almost consistent with the pathological tumor area, indicating good OS targeting ability. In clinical scenarios, if a resected OS specimen has a high fluorescence signal in the marginal area, this may indicate a positive margin and insufficient excision. The rapid pathological diagnosis of OS by the B7H3‐IRDye800CW probe does not need intravenous injection, so there is no need for tedious steps such as toxicity assessment, thus, B7H3‐IRDye800CW could be rapidly applied for the rapid pathological diagnosis of OS in the clinic.

There are some limitations in this study. Due to the small body size of the mouse, thoracoscopic resection, which is commonly used in clinical practice, cannot be used in the resection of mouse lung metastatic OS. OS specimens are too large (15 cm × 10 cm) for microtome and scanning equipment, thus, the specimen needed to be segmented into small pieces (2.5 cm × 2 cm) and spliced together after HE staining. There may be some tissue loss when segmenting. In addition, different tumor morphology may affect the result of specimen immersion, and some dead cavities may be more prone to accumulate probes and appear highlighted.

B7H3‐IRDye800CW has great translational potential. It is a conjugate of Omburtamab^[^ ^37^ ^]^ and IRDye800CW.^[^ ^38^ ^]^ The safety of both components has been verified in clinical trials,^[^ ^39^ ^]^ which means that the clinical translation time can be shortened greatly. Besides, the rapid pathological diagnosis of osteosarcoma specimen by B7H3‐IRDye800CW probe only needs in vitro incubation, so there is no need for tedious evaluation procedures including drug metabolism, pharmacokinetics, and toxicity.^[^ ^40^ ^]^ In summary, this study developed a new OS‐targeting NIR‐II probe, B7H3‐IRDye800CW, which showed excellent clinical value in intraoperative resection guidance and rapid pathological diagnosis of OS.

## 4 Experimental Section

### Cell Culture

OS cell lines 143B, MG63, KHOS, and U2OS were purchased from ATCC. 143B cell line expressing green fluorescent protein (143B‐GFP) and 143B cell line with expression of luciferase (143B‐LUC) were obtained from Beijing Zoman Biotechnology Co., Ltd. BMSC and HUVEC were purchased from National Infrastructure of Cell Line Resource. Cells were cultured with different mediums (143B, 143B‐GFP, 143B‐LUC, BMSC, HUVEC: DMEM; MG63 and KHOS: MEM; U2OS: McCoy's 5A), all culture medium was supplemented with 10% fetal bovine serum and 1% penicillin–streptomycin (Gibco), and cells were incubated at 37 °C in 5% CO_2_. For digestion procedures, cells were washed twice with PBS after discarding the culture medium. Subsequently, cells were digested with trypsin and resuspended in a culture medium.

### B7H3 Expression among Different Kinds of Cells

OS cell lines 143B, MG63, KHOS, U2OS, and normal cells BMSC and HUVEC were used in the experiment. For western‐blotting of B7H3, first, protease and phosphatase inhibitors were added into cell lysis buffer (Cell Signaling), the mixture was employed to lyse cells and extract protein, and after centrifugation at 12 000 g for 10 min, the supernatant was collected and a BCA protein concentration detection kit was used to obtain the protein concentration. Second, loading buffer was added to the supernatant and boiled for 10 min, after that, 25 µg total protein was loaded in each lane, and the protein was transferred onto polyvinylidene fluoride membrane after 10% sodium dodecyl sulfate‐polyacrylamide gel electrophoresis. Finally, the polyvinylidene fluoride membrane was incubated with primary antibody (Abcam, ab134161), horseradish peroxidase‐conjugated secondary antibody (Abcam, ab6721) in sequence, enhanced chemiluminescence substrate was added onto the polyvinylidene fluoride membrane and an imaging system (Bio‐rad) was used to record the bands.

For B7H3 immunofluorescence, the cells were inoculated on poly‐lysine treated cover slides, after the adhesion of cells, the cover slides were immersed in 4% paraformaldehyde for 10 min. Then, the cover slides were soaked in 95 °C alkaline urea buffer for 10 min, blocked with goat serum at room temperature for 30 min, after incubating in anti‐B7H3 antibody (Abcam, ab134161) at 4 °C overnight and rinsing with PBS for three times, Alexa Fluor 488 coupled goat anti‐rabbit IgG (Abcam, ab150077) was added onto the cover slides. At last, cells were co‐stained with 4′,6‐diamidino‐2‐phenylindole, and the cells were photographed by a fluorescence microscope (Leica). All experiments were repeated three times.

### B7H3 Expression among Different Types of Tissues

B7H3 immunohistochemical staining was used to reveal the B7H3 expression among OS and the most common surrounding normal tissues for the lack of peritumoral tissue. For OS B7H3 immunohistochemical staining, human primary OS specimens and lung metastatic OS specimens were chosen. For surrounding normal tissue staining, ten types of normal tissues that usually locate around OS were chosen: bone, muscle, periosteum, ligament, cartilage, nerve, growth plate, meniscus, bone marrow, and fat. For B7H3 immunohistochemical staining, briefly, fresh tissues were fixed with paraformaldehyde, dehydrated by ethanol, and embedded in wax, the specimens were sliced to 5 µm thickness and placed on slides, after dewaxing and hydration steps, the slides were incubated with anti‐B7H3 antibody (Abcam, ab134161) and horseradish peroxidase‐conjugated second antibody, respectively, diaminobenzidine solution was added onto the slides to visualize B7H3 expression and nucleus was stained with hematoxylin. Finally, the slides were observed with a microscope and analyzed with pathological software (Image‐Pro‐Plus, version 6.0.0.260), the detail could be found in previous literature.^[^ ^41^ ^]^

### Synthesis and Characterization of B7H3‐Targeted Fluorescent Probe

The B7H3‐targeted fluorescent probe was prepared by conjugating anti‐human B7H3 antibody with IRDye800CW. Briefly, the anti‐human B7H3 monoclonal antibody (Omburtamab, a drug that was tested in clinical trials)^[^ ^37^ ^]^ was dissolved in a phosphate buffer solution, and then, tenfold molar excess of IRDye800CW NHS ester was added to anti‐human B7H3 antibody solution, stirring the mixture in the dark at 4 °C for 8 h. After that, the mixture was purified using a PD‐10 desalting column (Cytiva), and unconjugated IRDye800CW NHS ester was eluted, the B7H3‐targeted fluorescent probe was concentrated to 0.5 mg mL^−1^ with a 30 000 Da molecular weight cutoff filter at 3000 g. The same procedures were employed to conjugate mouse IgG1 isotype (Thermofisher, 14‐4714‐85) with IRDye800CW, and isotype‐IRDye800CW was used as the isotype control probe.

B7H3‐IRDye800CW and IRDye800CW were dissolved in PBS and their absorption spectra were measured by a UV–vis spectrophotometer (Shimadzu UV‐2600), the emission spectra were determined by FLS980 (Edinburgh Instruments). The morphology of the targeted probe was observed by transmission electron microscopy (Tecnai 20), the average number of IRDye800CW molecules conjugated per anti‐B7H3 antibody was calculated through the absorbance curve of B7H3‐IRDye800CW (dissolved in 1:1 mixture of PBS and methanol), the affinity of anti‐B7H3 antibody and B7H3‐IRDye800CW at different concentration was determined by surface plasmon resonance.

### Cytotoxicity Evaluation of B7H3‐IRDye800CW

143B cells were inoculated onto a 96‐well plate. After cells were attached to the plate, anti‐B7H3 antibody, IRDye800CW, B7H3‐IRDye800CW, and ICG were added to the well plate to achieve a certain concentration (64, 128, 256 µg mL^−1^). After 24 h, the culture medium was discarded, the plate was rinsed with PBS, 10 µL CKK‐8 was added to each well, and a microplate reader was used to record the absorbance at 450 nm after 1 h.

### In Vitro Targeting Ability Evaluation

143B cells were digested with trypsin, resuspended in culture medium, and diluted to 500 000 cells per milliliter. The cells were transferred to a 6‐well plate with a cover glass. After cell adhesion, cell slides were fixed with 4% paraformaldehyde for 10 min. This experiment contained three groups: the B7H3‐IRDye800CW group, the isotype‐IRDye800CW group, and the blocking group. For the B7H3‐IRDye800CW group, cell slides were incubated with 10 µg mL^−1^ B7H3‐IRDye800CW in the dark for 2 h. For the isotype‐IRDye800CW group, cell slides were incubated with 10 µg mL^−1^ isotype‐IRDye800CW in the dark for 2 h. For the blocking group, the cell slides were pre‐incubated with 1 mg mL^−1^ Omburtamab for 2 h, followed by incubation with 10 µg mL^−1^ B7H3‐IRDye800CW in the dark for another 2 h. After incubation procedures, all slices were washed with PBS and then stained with 4′,6‐diamidino‐2‐phenylindole to visualize cell nuclei. Finally, a fluorescence microscope was used to observe the cell slides.

### NIR‐I and NIR‐II Fluorescence Image Capture

The NIR fluorescence capture was carried out in a dark room. A 792 nm laser was used to excite the probe, the NIR‐I fluorescence was captured by a complementary metal oxide semiconductor (CMOS) camera with a long‐pass filter that had a cutoff wavelength of 850 nm, the NIR‐II fluorescence was obtained by an InGaAs short‐wave infrared camera (Xenics Cheetah‐640CL TE3) with a filter wheel fixed in the front, the filter wheel had 1000, 1100, 1200, 1300, 1400, and 1500 nm filter that only NIR‐II fluorescence was allowed to pass through.

### GFP and BLI Imaging

GFP and BLI imaging were performed using the IVIS Spectrum In Vivo Imaging System (Caliper Life Sciences, Waltham, Massachusetts). The mouse was anesthetized before imaging. For GFP imaging, the mouse was placed into the IVIS Spectrum In Vivo Imaging System, and the excitation filter was set as 500 nm and the emission filter was set as 540 nm. For BLI imaging, 200 µL d‐luciferin potassium solution (15 mg mL^−1^) was intraperitoneally injected in advance, after 10 min, the BLI image was captured by IVIS Spectrum In Vivo Imaging System.

### Comparison of NIR‐I/II Fluorescence Imaging of Blood Vessels and Capillary Tube

For capillary tube fluorescent imaging, 10 µg mL^−1^ B7H3‐IRDye800CW was injected into a capillary tube with an inner diameter of 1 mm, the capillary tube was soaked in 2% fat emulsion with 5 mm depth. NIR‐I and NIR‐II fluorescence images were captured, exposure time was 30–1000 ms. The signal intensity profile was processed using LOWESS smoothing, and the FWHM was calculated. For mouse blood vessel imaging, BALB/C‐nude mice were obtained from Beijing Vital River Laboratory Animal Technology Co., Ltd. Mice were anesthetized and B7H3‐IRDye800CW was administered via tail vein (1 µg per gram mouse weight), NIR‐I and NIR‐II imaging were conducted on the tail vessel, the contrast between NIR‐I and NIR‐II was calculated through V/N, which was defined as the ratio of the fluorescence signal of blood vessel to the fluorescence signal to surrounding normal tissue.

### Establishment of Mouse Subcutaneous OS Model, Orthotopic OS Model, and Lung Metastatic OS Model

The BALB/C‐nude mice were purchased from Beijing Vital River Laboratory Animal Technology Co., Ltd. 143B‐GFP and 143B‐LUC cell lines were used in this experiment. For the subcutaneous OS model, 50 µL 143B‐GFP cell suspension (5 × 10^7^ cells per milliliter) was injected subcutaneously into the right back of mice. For the orthotopic OS model, 10 µL 143B‐LUC cell suspension (5 × 10^7^ cells per milliliter) was injected into the medullary cavity of the right tibia. The subcutaneous and orthotopic OS models were considered successful after observing rapid enlargement of soft tissue lump at the injection site, subsequent probe injection and NIR imaging were performed when the long diameter reached 0.6–1 cm. For the lung metastatic OS model, 50 µL 143B‐LUC cell suspension was injected into the tail vein. After 10–14 days, an IVIS Spectrum In Vivo Imaging System was used to conduct BLI imaging for the mice. The lung metastatic OS model could be considered successful when obvious BLI in the chest region was observed.

### In Vivo Comparison of NIR‐I/II Fluorescence Imaging

Mouse subcutaneous OS model and orthotopic OS model were used in the experiment (*n* = 5). B7H3‐IRDye800CW was systematically administrated to the OS‐bearing mice. After 24 h, the probe was excited by a 792 nm laser. NIR‐I fluorescence image was captured by a CMOS camera (850 nm). NIR‐II fluorescence image was recorded by an InGaAs short‐wave infrared camera (1000 nm, chosen based on the results of the capillary tube and mice tail vessel imaging). NIR‐I and NIR‐II fluorescence images were processed with Image J software (version 1.52, NIH, USA).

### The Relationship between the Concentration of B7H3‐IRDye800CW and NIR‐I/II Fluorescence Intensity

Briefly, B7H3‐IRDye800CW was diluted to different concentrations (2, 4, 6, 8, 10, and 12 µg mL^−1^) and added to a black 96‐well plate. NIR‐I and NIR‐II fluorescence images were captured, and the relationship between the concentration of B7H3‐IRDye800CW and NIR‐I/II fluorescence intensity was analyzed.

### In Vivo Targeting Ability and Biodistribution Evaluation

Mouse subcutaneous OS model was used in the experiment. The experiment contained three groups: the B7H3‐IRDye800CW group, the isotype‐IRDye800CW group, and the blocking group (*n* = 5). For B7H3‐IRDye800CW group, B7H3‐IRDye800CW was administered through tail vein at a dose of 1 µg per gram of body weight; for isotype‐IRDye800CW group, isotype‐IRDye800CW was injected into the tail vein (1 µg g^−1^); for blocking group, Omburtamab was administered previously (60 µg g^−1^). After 24 h, B7H3‐IRDye800CW was intravenously injected into the OS‐bearing mice (1 µg g^−1^). At 2, 4, 8, 12, 24, 48, and 72 h after administration of the probe, mice were first anesthetized with 2.5% isoflurane (2.5 L min^−1^), then NIR‐I (850 nm) and NIR‐II (1000 nm) fluorescence images were captured. The fluorescence intensity was calculated by Image J, and TBR was defined as the ratio of tumor area fluorescence intensity to the peritumor area fluorescence intensity.

As for in vivo organ distribution, mice from three groups were sacrificed by overdose anesthesia 24 h after probe administration (*n* = 3). Then, mice were dissected by scalpel, and subcutaneous OS and major organs, including heart, liver, spleen, lung, kidney, intestine, and muscle, were taken out. NIR‐II fluorescence images (1000 nm) were captured, and the organs (heart, liver, spleen, lung, and kidney) were immersed in 4% paraformaldehyde for further pathological analysis. The fluorescence intensity was measured by Image J.

### In Vivo Targeting Accuracy Evaluation

Mouse subcutaneous OS model, orthotopic OS model, and lung metastatic OS model were used in the experiment (*n* = 3). B7H3‐IRDye800CW was intravenously injected into the OS‐bearing mice. After 24 h, GFP imaging was performed by an IVIS Spectrum In Vivo Imaging System on a mouse subcutaneous OS model (143B‐GFP cell line). For orthotopic OS and lung metastatic OS model, BLI imaging was recorded 10 min after intraperitoneal administration of d‐luciferin potassium solution. NIR‐II images were obtained following the procedure described earlier. Finally, the tumor areas shown by GFP or BLI were compared with the tumor area labeled by the B7H3‐IRDye800CW targeting probe.

### NIR‐II Fluorescence Guided OS Resection

The mouse orthotopic OS model and lung metastatic OS model were used in the experiment (*n* = 5). Before the operation, mice were anesthetized by isoflurane, d‐luciferin potassium was administrated every 30 min, and the OS was first resected with naked eyes till no tumor could be found nakedly. Then, BLI and NIR‐II fluorescence imaging were used to find out if there was remaining OS, if there was residual OS, NIR‐II fluorescence imaging (1000 nm) was used to assist in the removal of the remaining OS, after the NIR‐II fluorescence‐assisted excision, BLI imaging was conducted to confirm whether the tumor was completely removed. For the lung metastatic OS model, the chest was opened previously to observe the lung metastases, and then, NIR‐II fluorescence imaging (1000 nm) was used to assist in the location and removal of the lung metastatic OS.

### In Vitro Incubation and Imaging of Human OS Samples

Five OS patients' resected specimens were obtained. All diagnoses were confirmed by preoperative biopsy. All patients underwent surgery after receiving first‐line neoadjuvant chemotherapy. Freshly excised specimens were first cut in half (*n* = 5), a 3 mm thick specimen was sawed off from the half specimen, the specimen was rinsed with saline to remove the sawdust, and then, the specimens were immersed into Kerbs–Henseleit's solution containing 2% fetal bovine serum. After 5 min, the specimens were incubated in B7H3‐IRDye800CW solution (PBS solution containing 50 µg mL^−1^ B7H3‐IRDye800CW and 30% PEG‐300) for 30 min. Finally, the specimens were washed in PBS solution containing 30% PEG‐300 three times, 2 min at a time. The specimens were then dried with absorbent paper, and NIR‐II fluorescence images (1000 nm) of the specimens were captured.

The specimens were cut into 2.5 cm × 2 cm tissue blocks by hard tissue microtome. The tissue blocks were fixed with 4% paraformaldehyde, decalcified by formic acid, embedded in wax, and sliced into 5 µm thickness. The slices were stained by HE and scanned by a pathological section scanner, splicing the tissue slice into a whole. The pathological tumor area was compared with the tumor area depicted by NIR‐II fluorescence imaging.

### Statistical Analysis

Image J software was used to analyze NIR fluorescence intensity quantitatively and convert a 16‐bit grayscale image to pseudo color. Quantitative data was expressed as mean ± standard deviation (SD). Student's *t*‐test was used to compare whether there was a difference between two groups, one‐way ANOVA test was used to evaluate the difference among three or more groups. *p* < 0.05 was defined as statistically significant. *, **, *** and **** represent *p* < 0.05, *p* < 0.01, *p* < 0.001, *p* < 0.0001, respectively.

### Ethics Statement

The human experiment was conducted in compliance with the Declaration of Helsinki and was approved by the Ethics Committee of Peking University People's Hospital (2023PHB433‐001). Participants gave informed consent to participate in the study. The animal experiments followed the 3R principles and the guidelines of the Institutional Animal Care and Use Committee of the Beijing Municipal Science & Technology Commission. Animal experiments were approved by the Institutional Animal Care and Use Committee of Peking University People's Hospital (2023PHE121).

## Conflict of Interest

The authors declare no conflict of interest.

## Supporting information

Supporting Information

## Data Availability

The data that support the findings of this study are available from the corresponding author upon reasonable request.
