# Supplementary material for: Intraoperative Resection Guidance and Rapid Pathological Diagnosis of Osteosarcoma using B7H3 Targeted Probe under NIR‐II Fluorescence Imaging
Source: Adv Sci (Weinh). 2024 Mar 19;11(33):2310167. doi: 10.1002/advs.202310167 (PMC11434027; doi:10.1002/advs.202310167)
Supplement: Supplementary file 1 — Supporting Information [file ADVS-11-2310167-s001.pdf]

ADVANCED SCIENCE
Open Access

Supporting Information

for *Adv. Sci.*, DOI 10.1002/advs.202310167

Intraoperative Resection Guidance and Rapid Pathological Diagnosis of Osteosarcoma using B7H3 Targeted Probe under NIR-II Fluorescence Imaging

Fanwei Zeng, Changjian Li, Han Wang, Yueqi Wang, Tingting Ren, Fangzhou He, Jie Jiang, Jiuhui Xu, Boyang Wang, Yifan Wu, Yiyang Yu, Zhenhua Hu, Jie Tian*, Shidong Wang* and Xiaodong Tang**

Intraoperative Resection Guidance and Rapid Pathological Diagnosis of Osteosarcoma using B7H3 Targeted Probe under NIR-II Fluorescence Imaging

Fanwei Zeng, Changjian Li, Han Wang, Yueqi Wang, Tingting Ren, Fangzhou He, Jie Jiang, Jiuhui Xu, Boyang Wang, Yifan Wu, Yiyang Yu, Zhenhua Hu,* Jie Tian,* Shidong Wang,* Xiaodong Tang*

F. Zeng, H. Wang, T. Ren, F. He, J. Jiang, J. Xu, B. Wang, Y. Wu, Y. Yu, S. Wang, X. Tang

Department of Musculoskeletal Tumor & Beijing Key Laboratory of Musculoskeletal Tumor

Peking University People's Hospital, Beijing 100044, China.

E-mail: tang15877@126.com; stonewang@bjmu.edu.cn

C. Li, J. Tian

School of Engineering Medicine & Key Laboratory of Big Data-Based Precision Medicine

Beihang University, Ministry of Industry and Information Technology, Beijing 100191, China

Y. Wang, Z. Hu, J. Tian

CAS Key Laboratory of Molecular Imaging, Beijing Key Laboratory of Molecular Imaging, Institute of Automation, Chinese Academy of Sciences, Beijing, 100190, China.

E-mail: jie.tian@ia.ac.cn; zhenhua.hu@ia.ac.cn

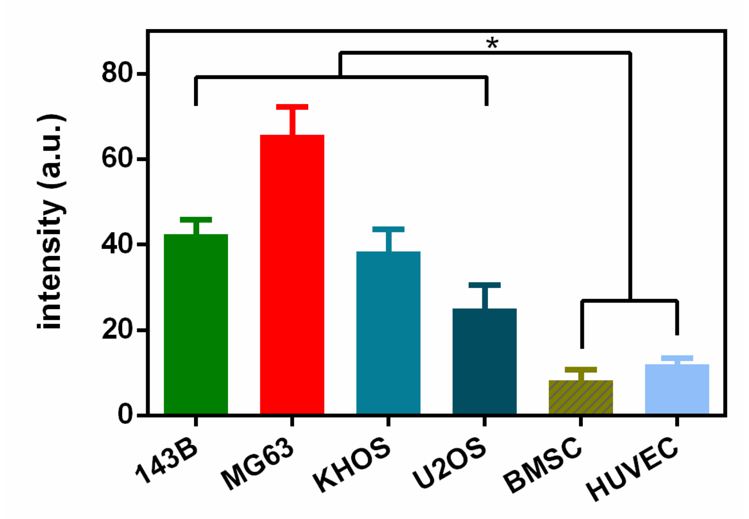

Figure S1. Quantitative analysis of B7H3 immunofluorescence results of 143B, MG63, KHOS, U2OS, BMSC and HUVEC. (* P<0.05)

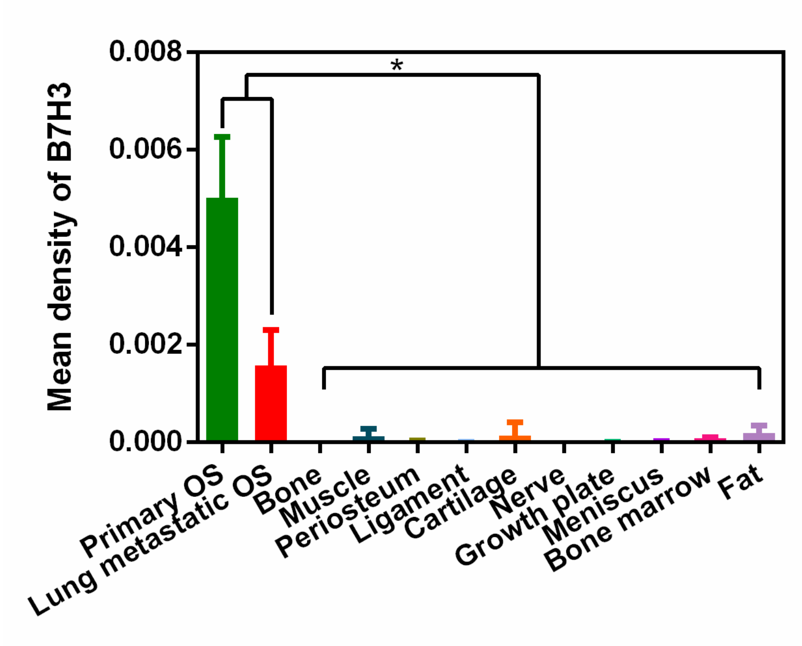

Figure S2. Mean density of human primary OS, lung metastatic OS and normal tissues around osteosarcoma-prone areas including bone, muscle, periosteum, ligament, cartilage, nerve, growth plate, meniscus, bone marrow and fat. (* P<0.05)

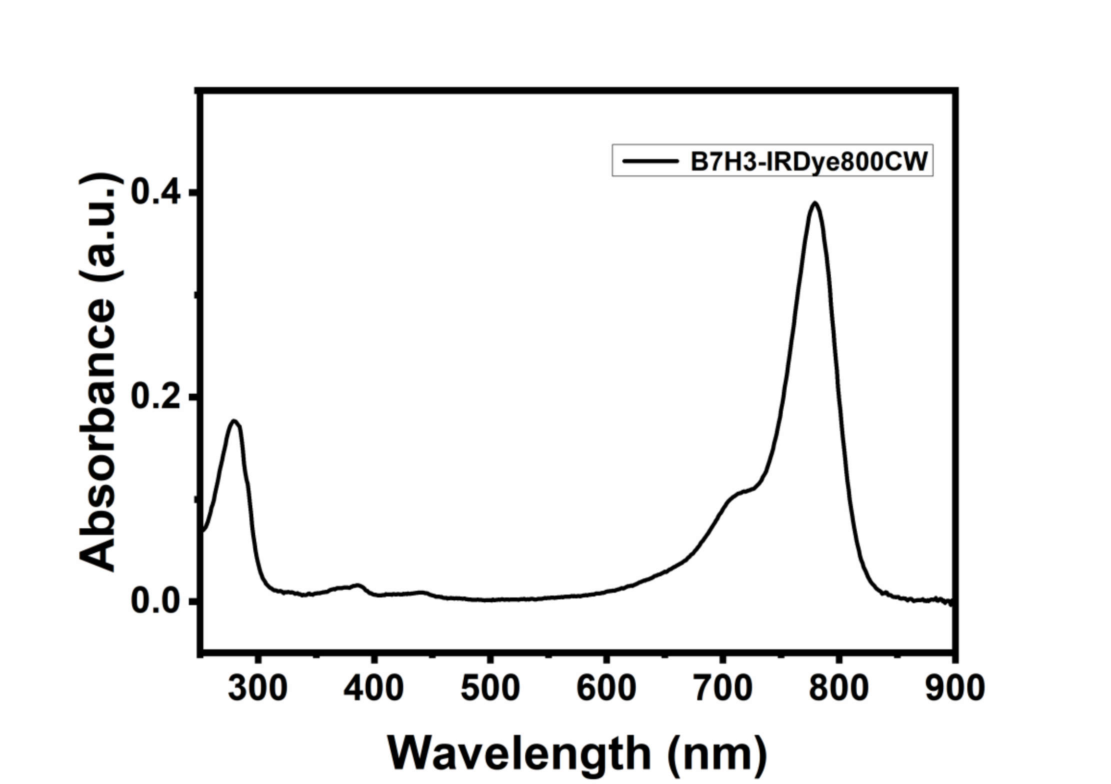

Figure S3. The UV-visible-NIR absorption spectrum of B7H3-IRDye800CW in 1:1 mixture of PBS and methanol.

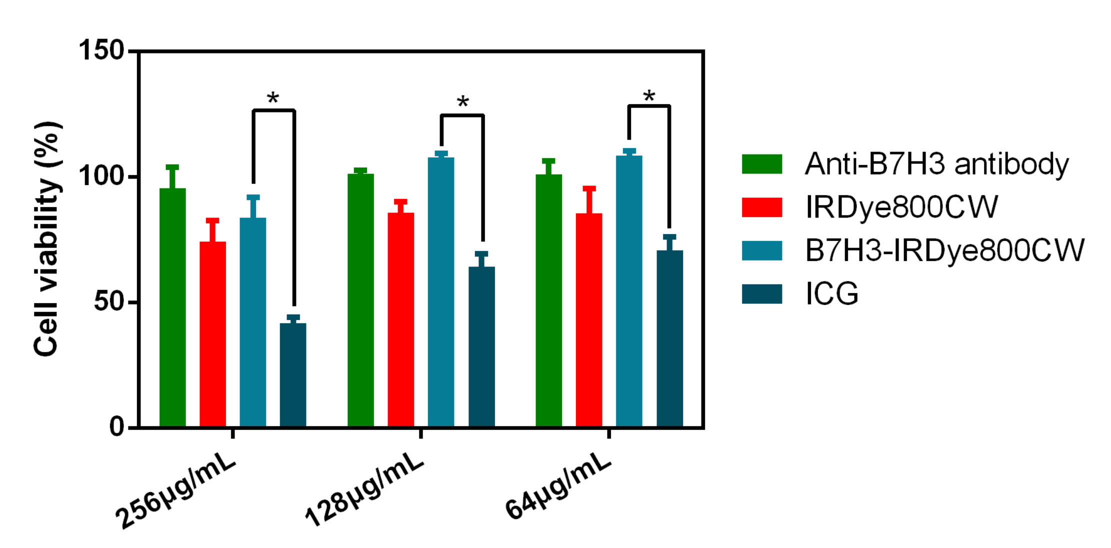

Figure S4. Cell viability of 143B cell lines after incubating with 64, 128 and 256 μg/mL anti-B7H3 antibody, IRDye800CW, B7H3-IRDye800CW and ICG for 24 h.

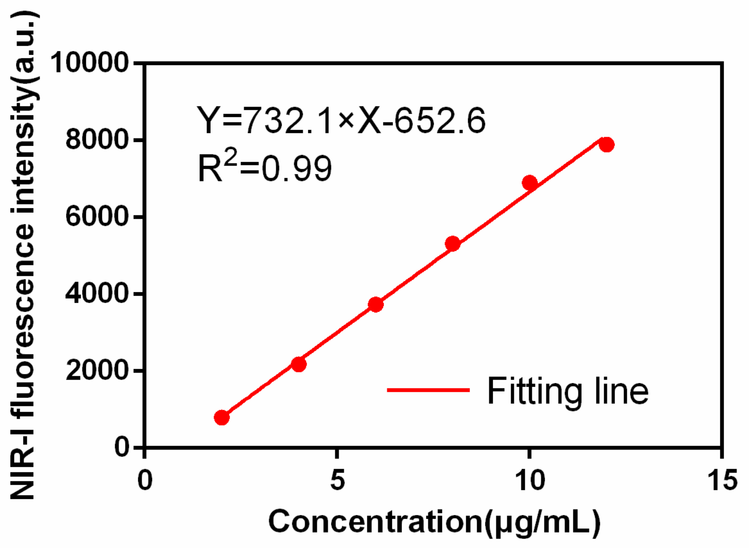

Figure S5. The NIR-I fluorescence intensity of B7H3-IRDye800CW is linearly related to the concentration.

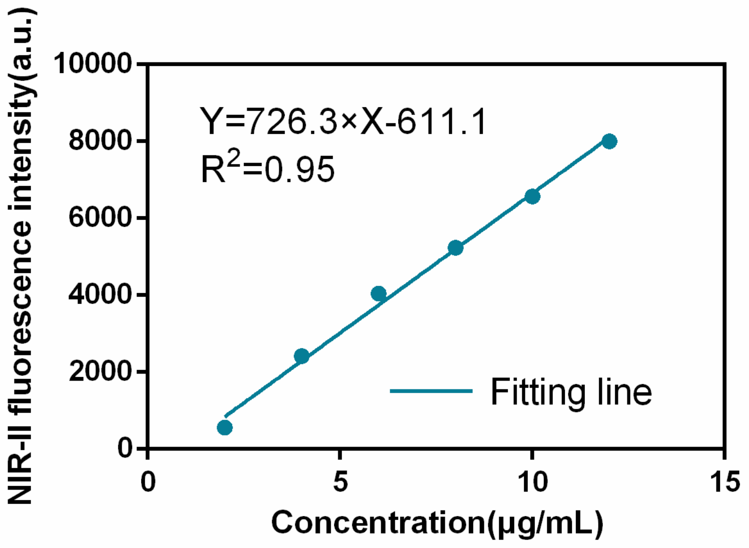

Figure S6. The NIR-II fluorescence intensity of B7H3-IRDye800CW is linearly related to the concentration.

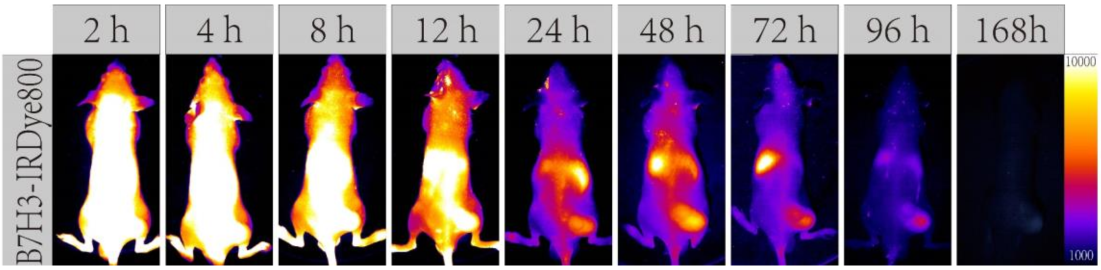

Figure S7. Continuous observation of OS-bearing mouse after injection of B7H3-IRDye800CW under NIR-II fluorescence imaging (2-168 h).

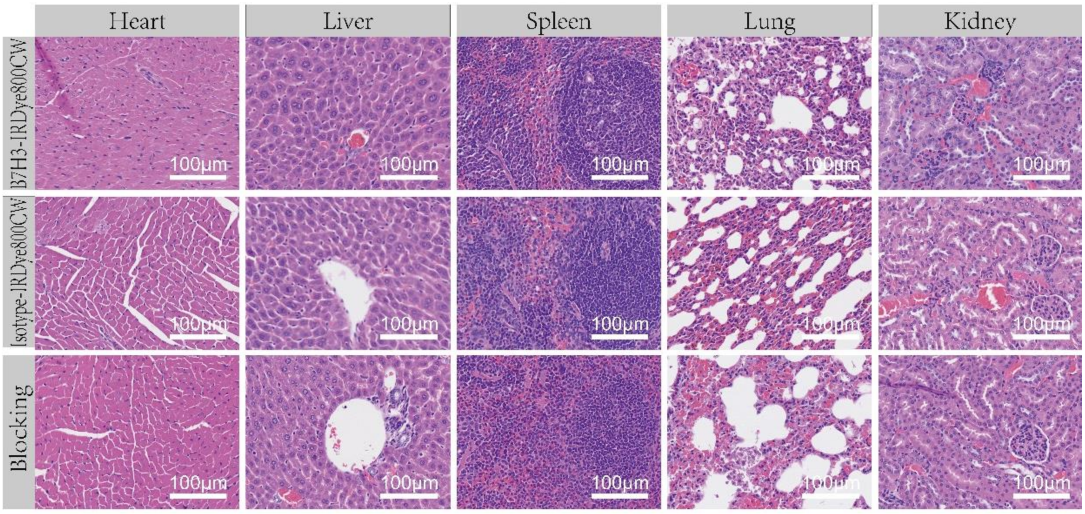

Figure S8. Histological evaluation of major organs (heart, liver, spleen, lung, kidney). Scale bar: 100 μm

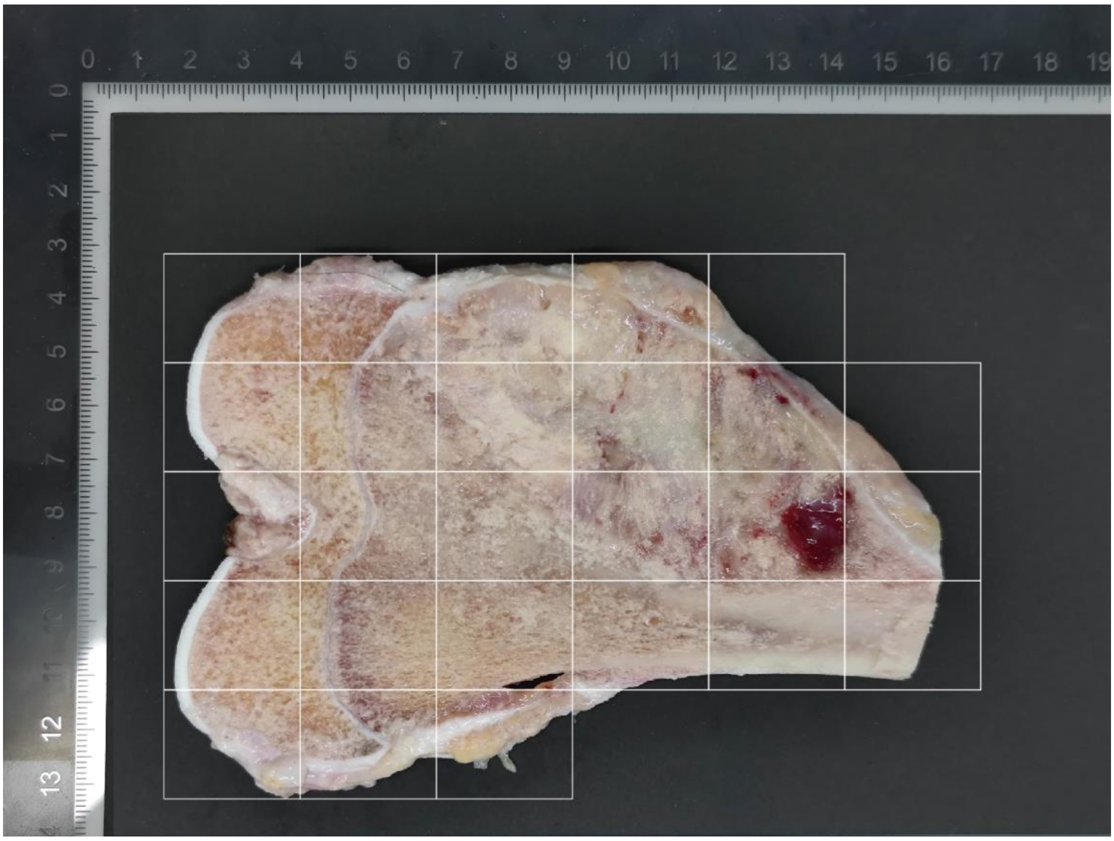

Figure S9. The segmentation method for human OS specimen (No. 4 OS patient).

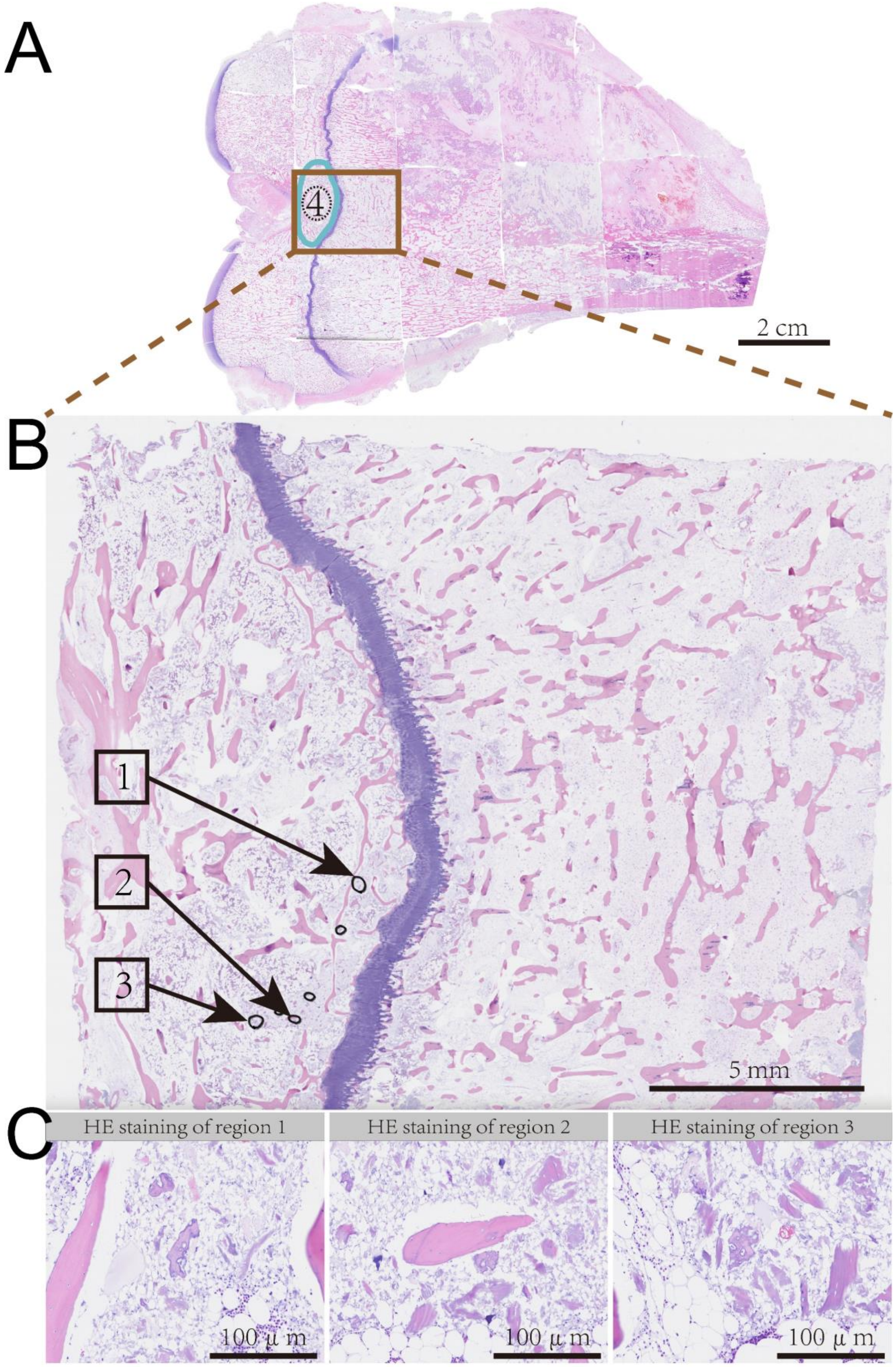

Figure S10. A) Complete HE staining of the specimen, B) OS microinvasion lesions in

region 4, the black line circled areas are all OS microinvasions. C) Microscopic images of region 1, 2 and 3 in B). Scale bar was labeled on the figure.
